# Temporal characterisation of the organ-specific *Rhipicephalusmicroplus* transcriptional response to *Anaplasma marginale* infection

**DOI:** 10.1016/j.ijpara.2011.03.003

**Published:** 2011-07

**Authors:** Ricardo F. Mercado-Curiel, Guy H. Palmer, Felix D. Guerrero, Kelly A. Brayton

**Affiliations:** a Program in Vector-Borne Diseases, Department of Veterinary Microbiology and Pathology and Paul G. Allen School for Global Animal Health, Washington State University, Pullman, WA 99164-7040, USA; b USDA-ARS, Knipling-Bushland U.S. Livestock Insects Research Laboratory, 2700 Fredericksburg Rd., Kerrville, TX 78028, USA

**Keywords:** Tick-borne disease, Ixodid, Rickettsial, Gene expression, Microarray

## Abstract

Arthropods transmit important infectious diseases of humans and animals. Importantly, replication and the development of pathogen infectivity are tightly linked to vector feeding on the mammalian host; thus analysis of the transcriptomes of both vector and pathogen during feeding is fundamental to understanding transmission. Using *Anaplasma marginale* infection of *Rhipicephalusmicroplus* as the experimental model, we tested three hypotheses exploring the temporal and organ-specific nature of the tick midgut and salivary gland transcriptomes during feeding and in response to infection. Numerous *R. microplus* genes were regulated in response to feeding and were differentially regulated between the midgut and salivary gland; additionally, there was a progression in regulated gene expression in the salivary gland over time. In contrast, relatively few tick genes were specifically regulated in response to *A. marginale* infection and these genes were predominantly annotated as hypothetical or were of unknown function. Notable among the genes with informative annotation was that several ribosomal proteins were down-regulated, suggesting that there may be a corresponding decrease in translation. The hypotheses that *R. microplus* midgut and salivary gland genes are differentially regulated and that the salivary gland transcriptome is dynamic over time were accepted. This is consistent with, and important for understanding the roles of, the two organs, the midgut serving as an initial site of uptake and replication while the salivary gland serves as the final site of replication and secretion. The nominal effect of *A. marginale* on the tick transcriptome in terms of numbers of regulated genes and fold of regulation supports the view that the vector–pathogen relationship is well established with minimal deleterious effect on the tick. The small set of predominantly hypothetical genes regulated by infection suggests that *A. marginale* is affecting a novel set of tick genes and may provide new opportunities for blocking transmission from the tick.

## Graphical abstract

**Graphical abstract:**

**Figure:**
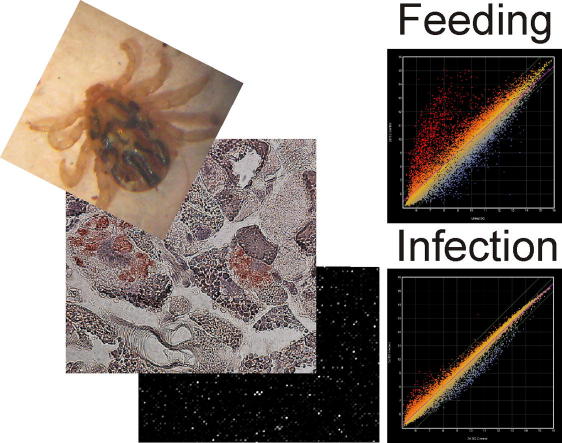

****Highlights**:**

► Analysis of *Rhipicephalus microplus* tick gene expression during feeding. ► A massive organ-specific transcriptional response to tick feeding is confirmed. ► Analysis of tick response to infection with the cattle pathogen *Anaplasma marginale*. *► Anaplasma marginale* exerts a minimal effect on the tick transcriptome.

## 1 Introduction

Microbial transmission to a mammalian host requires a minimal infectious dose, encompassing both absolute number and relative infectivity of the pathogen. For those agents transmitted by arthropods, the causes of some of the most prevalent and debilitating diseases of humans and animals, replication and acquisition of infectivity occur within the invertebrate vector prior to transmission, a very different cellular environment from that of the mammalian host (Pal and Fikrig, 2003; Nelson et al., 2008; Ramabu et al., 2010). The most successful vector-borne pathogens have evolved to coordinate their replication with the feeding behaviour of the vector, thus ensuring that the infective dose is optimised at the opportune moment. The vector itself undergoes dramatic phenotypic changes during feeding on the mammalian host, from rapid growth to organ development to molting between life stages, all of which are linked to changes in gene expression and with which pathogen replication and development of infectivity is tightly linked (Francischetti et al., 2009; Rodriguez-Valle et al., 2010). Understanding the linkages between vector gene expression and pathogen gene expression, resulting in development of an infective dose, is fundamental to understanding the transmission biology of vector-borne microbial pathogens.

We have addressed these basic questions by studying tick transmission of the rickettsial pathogen *Anaplasma marginale*. Several genera of ixodid ticks, including *Dermacentor*, *Hyalomma* and *Rhipicephalus* spp., acquire *A. marginale* by feeding on infected ruminants. Following sequential colonisation and replication in the midgut epithelium and salivary glands, the bacteria successfully transmit to additional susceptible individuals (Kocan et al., 1985, 1992; Shkap et al., 2009). Replication within the salivary gland at the time of transmission feeding is critical for successful transmission and is intimately tied to marked changes in the tick salivary gland. Notably, transmission efficiency increases with the duration of this transmission feeding, indicative of the close adaptation of the pathogen to tick feeding behaviour (Kocan et al., 1985).

In addressing this vector–pathogen interaction, we propose and test three linked hypotheses. The first is that the regulation of the tick transcriptome is organ specific: the midgut transcriptome is unique during feeding and during acquisition of *A. marginale* compared with the salivary gland. This distinction is relevant as the two organs serve very different roles in the transmission biology of *A. marginale* with early survival and replication within the midgut epithelium, composed of highly phagocytic cells, required for initial colonisation while a second round of replication in the salivary gland acini, composed of highly secretory cells, is required for transmission of an infective dose in the saliva (Kocan et al., 1992; Lohr et al., 2002). Importantly, both the midgut epithelium and salivary glands have been identified as separate and distinct barriers for transmission of *A. marginale* and thus represent two potential sites where transmission could be blocked (Ueti et al., 2007). The second hypothesis to be tested is that the salivary gland transcriptome is temporally dynamic. Initiation of tick attachment and feeding involves secretion of a virtual pharmacopeia including lytic enzymes, anticoagulants and inhibitors of the mammalian innate immune and nociceptive systems (Francischetti et al., 2009). Concomitantly, the acini provide an environment where *A. marginale* replicates >100-fold and are secreted into the saliva. Prior studies show that duration of feeding is a critical component of transmission efficiency, with increased efficiency positively correlated with time of tick feeding (Kocan et al., 1992).

The third hypothesis to be tested is that *A. marginale* colonisation does not significantly modulate the tick salivary gland transcriptome. This hypothesis is based on observations by ourselves and others that tick infection does not impart a significant fitness cost on the vector. This is in contrast to other bacterial and protozoan pathogens that have dramatic effects on the success of tick attachment, engorgement and survival (Ouhelli et al., 1987; Ross and Levin, 2004; Dong et al., 2006). *Anaplasma marginale*, similar to other tick-borne pathogens in the Family Anaplasmataceae, is believed to have evolved from an arthropod-specific bacterium with relatively late adaptation to specific niches in mammalian hosts (Sallstrom and Andersson, 2005; Ramabu et al., 2010). Consequently, we predict that *A. marginale* is well adapted to its tick vector and utilises the normal signalling pathways of the feeding tick with few, if any, effects on the salivary gland transcriptome over time. In this manuscript, we report the testing of these three hypotheses and present the results in the context of vector–pathogen–mammalian host interaction at the time of transmission.

## 2 Materials and methods

### 2.1 Tick feedings and experimental design

Adult male ticks of the *Rhipicephalus microplus* La Minita stock (originally from Texas, USA) were used in the study (Futse et al., 2003). Molting nymphs were collected from the bovine host and allowed to complete the molt while being held at 26 °C, 95% humidity. Unfed adult ticks were dissected within 48 h of molting. Cohorts of unfed adult ticks were applied to either an *A. marginale* St. Maries strain-infected Holstein calf or, as a control, an age-matched uninfected calf and allowed to feed for 2, 6 or 9 days. Animal experiments were approved by the Institutional Animal Care and Use Committee at Washington State University, USA, in accordance with institutional guidelines based on the U.S. National Institutes of Health (NIH) Guide for the Care and Use of Laboratory Animals. Infection was established and monitored by Giemsa-stained blood smear as previously described (Futse et al., 2003). Ticks were then removed and dissected for collection of salivary glands or midguts (at 2 days only). The 10 treatment groups are listed in Table 1. Each treatment group was analysed as three biological replicates composed of 90 pooled tissue samples collected at each time point/condition and stored in Trizol. Single tick tissues were sampled for infection level and rate determinations, as described below.

### 2.2 RNA treatment and microarray

Ten micrograms of high-quality DNase treated-total RNA from each tissue pool was used for double strand cDNA synthesis using the SuperScript Double-Stranded cDNA Synthesis Kit (Invitrogen) and labelled with Cy3 using the One-Color DNA Labelling Kit (Roche NimbleGen, USA) according to the manufacturer’s recommendations. A Roche NimbleGen 12×135K HD2 gene expression microarray was designed using the *R. microplus* expressed sequence tag (EST) database, *R. microplus* Gene Index Version 2.1 (BmiGI V2.1) (Wang et al., 2007; Guerrero et al., 2008). BmiGI V2.1 contains 14,586 unique sequences including 9,851 tentative consensus (TC) sequences and 4,735 singletons that were generated from a total of 42,852 sequences. Eight 60 mer probes were designed for 14,447 unique ESTs which were used for the microarray manufacture. Array hybridisation employed the Hybridization Kit (Roche NimbleGen). Briefly, 2 μg Cy3-labelled cDNA were resuspended in 3.3 μl Sample Tracking Control and 8.7 μl of master mix, and incubated at 95 °C for 5 min. Slides were hybridised at 42 °C for 16 h followed by washing using the Wash Buffer Kit (Roche NimbleGen) and Array Processing Accessories (Roche NimbleGen) as recommended. One-Color array scanning was performed in a GenePix 4000B scanner (Molecular Devices, USA) using default settings, obtaining the desired histogram with 1e-5 normalised counts at 65,000 saturation (intensity level).

### 2.3 Microarray analysis

NimbleScan software (http://www.nimblegen.com/products/software/) was used to extract the raw intensity values. Several quality controls of the experimental data were carried out at different stages of the analysis: first, visual and software-generated Sample Tracking analysis confirmed the experimental integrity, sample identity and lack of cross-contamination on each array. Second, the quality of the data was verified by the Experimental Metrics Report provided by the software, evaluating parameters such as Uniformity Mean for the mean signal intensity of all probes in each uniformity block and the Uniformity Coefficient of Variation for the coefficient of variation of the block uniformity means. The Mean Random for the mean signal intensity of the random control features present on the array which have the same length and guanine–cytosine (GC) characteristics as the experimental probes on the array was used to estimate the amount of non-specific binding in the hybridisation.

Additional quality controls, statistical and advanced analyses of the data were carried out using the R-project statistical environment (http://www.r-project.org) and Bioconductor (Gentleman et al., 2004). These analyses included: (i) box plot, histogram and cluster analysis of raw data; (ii) Robust Multi-array Average (RMA) normalisation (background correction, quantile normalisation, log transformation, summarisation); (iii) box plot, histogram and cluster analysis of RMA normalised data; (iv) comparisons between groups of interest to determine the statistically significant ratios or fold-change values (*P* < 0.05) determined by eBayes with Benjamini & Hochberg’s False Discovery Rate (FDR) correction; and (v) Principal Component Analysis (PCA). The microarray data have been deposited in the NCBI GEO public database (Accession No.: GSE21690).

### 2.4 Quantitative reverse transcription (RT)-PCR (qPCR)

The expression profile was validated for all treatment groups for three randomly selected genes amongst those found to be regulated by infection at day 9 in salivary glands. TaqMan quantitative PCR (qPCR) was performed using aliquots from the same RNA samples used for microarray hybridisations (Futse et al., 2003). Random primed, single stranded cDNA was synthesised using the SuperScript III First-Strand Synthesis SuperMix for qRT-PCR Kit (Invitrogen), and used as a template for qPCR using iQ-Supermix (BioRad, Hercules, CA, USA) reagents. The sets of primers and TaqMan probes used for each selected gene were as follows: CK176798, forward 5′-TCA CGT CAC ATG ATC CCA CCT TGT-3′ and reverse 5′-AAA GAA ACA CTG CTC GTT CAC GCC-3′ and probe 5′-CCG AAG TAG TTG CTT ACG AAA CTG CTG C-3′; TC17710, forward 5′-TGT CGG GCT GTC TTT CAC AGT TCA-3′ and reverse 5′-TAC AGC GTG GAA CGA GAG GAC AAA-3′ and probe 5′-ATG CGT TAC TTG TTG GTC TGC CGT GT-3′; TC24862, forward 5′-AGC GGG AAA GGG AAG AAC AAA GGA-3′ and reverse 5′-TCT TCA TCG CTG TCA TCT GCC TGT-3′ and probe 5′-AAA GGC AGA GGC AAG GGA AGC AGA AA-3′. The mRNA levels were normalised against *R. microplus* actin, which was quantified using the following oligonucleotides: forward 5′-AAG CGT GGT ATC CTC ACC CTG AAG TA-3′ and reverse 5′-AGG TCT CGA ACA TGA TCT GCG TCA-3′ and TaqMan probe 5′-ATG GAG AAG ATC TGG CAC CAC ACC TT-3′. The qPCR conditions were 95 °C for 5 min, 58 cycles of 95 °C for 35 s, 59 °C for 53 s and 72 °C for 70 s, final extension at 72 °C for 7 min and holding at 4 °C. All assays were done in triplicate. The relative transcript levels of the three genes in the infected or fed group and its respective uninfected or unfed control group were determined by the ΔΔCt method (Livak and Schmittgen, 2001). Data are presented as 2^−ΔΔCt ± S.D.^ (*P* < 0.05) determined by Student’s *t*-test. The presence or absence of correspondence between results from qPCR and microarray were analysed for each comparison.

### 2.5 Determination of *A. marginale* infection rates/levels in single tick tissues

Genomic DNA extracted from dissected midgut and salivary glands of single ticks was analysed for the level of *A. marginale* infection by qPCR for the single copy gene *msp5* as previously described (Scoles et al., 2007). Briefly, forward 5′-CTT CCG AAG TTG TAA GTG AGG GCA-3′ and reverse 5′-CTT ATC GGC ATG GTC GCC TAG TTT-3′ primers together with a TaqMan probe (5′-GCC TCC GCG TCT TTC AAC AAT TTG GT-3′) were used to enumerate *A. marginale* copy number. The qPCR assay was performed using the iQ-Supermix (BioRad, Hercules, CA, USA) as follows: 95 °C for 5 min, 55 cycles of 95 °C for 35 s, 59 °C for 53 s and 72 °C for 70 s, final extension at 72 °C for 7 min and holding at 4 °C. Triplicate amplification of the unknown DNA samples was carried out simultaneously with serial dilutions of cloned *msp5*, and the results are presented as mean number of bacteria per total organ (±S.D.). Samples showing a quantifiable amount of *A. marginale* were scored as positive and used to determine the infection rate for the group of singly collected tick tissues.

## 3 Results

### 3.1 *Anaplasma marginale* infection rates/levels in *R. microplus* ticks

Cohorts of *R. microplus* ticks were exposed to *A. marginale* levels ranging from 10^7^ to 10^9^ organisms/ml of blood during the 9 day feeding period. The level and the rate of infection was determined by qPCR for midgut or salivary gland pairs obtained from single ticks from each group (Table 1). The highest infection rate was 89% observed at day 2 in the midgut. The salivary gland samples showed both an increasing infection rate and level throughout the feeding period, culminating at day 9 with 65% of the ticks showing an infection of ∼6X10^5^ bacteria/salivary gland pair. Uninfected control groups were uniformly negative.

### 3.2 Differential gene expression upon infection or feeding

Expression analysis demonstrated that 78% of sequences included on the microarray were expressed in at least one sample collected in the study. As the BmiGI V2.1 was developed from ticks at different life stages exposed to a variety of stimuli, not all sequences would be expected to be expressed (Wang et al., 2007). The *R. microplus* gene expression profile of 14,447 sequences was analysed from midgut and salivary glands, comparing time point matched infected versus uninfected ticks as well as fed ticks compared with unfed ticks (Table 2).

The response to acquisition feeding was rapid and dramatic with as many as 34% of the genes demonstrating a regulatory effect at day two of feeding. Interestingly, more genes were down-regulated in response to feeding than were up-regulated, however the magnitude was typically greater for up-regulated genes than for down-regulated genes. The response to infection showed a more complicated picture with a rapid regulation of genes in the midgut by day 2, but a much slower evolution of statistically significant regulation within the salivary gland, with only 106 genes being up-regulated and 40 genes being down-regulated at day 9 (Table 2). The lack of observed response in the salivary glands at days 2 and 6 likely reflects both the lower infection rate and level observed at these time points, i.e. the response cannot be detected amongst the background of uninfected ticks in these populations. Both the magnitude and number of genes regulated in response to infection were much smaller than in response to feeding.

### 3.3 Validation of microarray data by qPCR

**Fig. 1:**
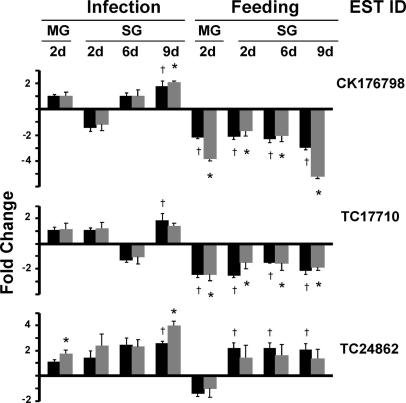
Quantitative (q)PCR validation of microarray data for randomly selected genes of *Rhipicephalus microplus*. The expression profile was validated throughout all of the time points/treatment groups for three genes of unknown function, CK176798, TC17710 and TC24862, which were randomly selected within the set of genes identified by microarray to be up-regulated in salivary glands at day (d) 9 of *Anaplasma marginale* infection. Bars represent relative transcript levels between the infected or fed group and its respective uninfected or unfed control group. Data for qPCR are 2^−ΔΔCt ± S.D.^ (gray bars). Microarray data (black bars) are presented as fold-change values. ^∗^ And ^†^ indicate statistical significance as described in the Section 2.4. MG, midgut; SG, salivary glands; ESTID, expressed sequence tag identification number.

qPCR was used to verify the microarray expression data set. The expression profile was validated for each time point and tissue for three randomly selected genes (CK176798, TC17710 and TC24862) that were found to be up-regulated in the salivary gland at day 9 of *A. marginale* infection (Fig. 1). qRT-PCR analysis confirmed that these genes were significantly differentially regulated (*P* < 0.05) in most of the pairwise comparisons as indicated by the microarray data. Two exceptions were TC17710 in the salivary gland at day 9 in response to infection and TC24862 in the salivary gland at all time points during feeding. Overall, there was concordance in the direction of the fold-change observed between the microarray data and the qRT-PCR assays and reasonable agreement in the magnitude of the fold-change values (Fig. 1).

### 3.4 *Rhipicephalus microplus* differential gene expression due to infection

**Fig. 2:**
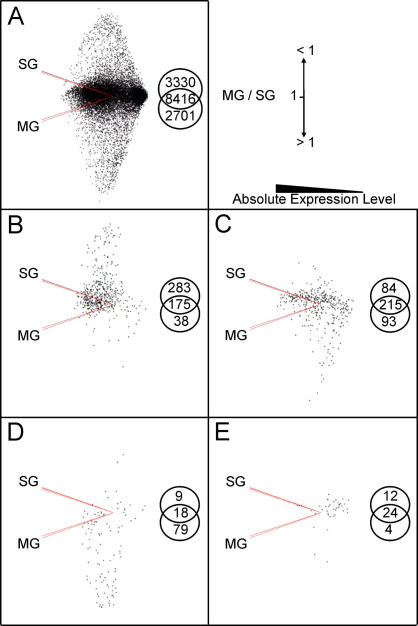
Principal Component Analysis (PCA) of *Rhipicephalus microplus* genes regulated by infection with *Anaplasma marginale*. PCA with unfed midgut (MG) and salivary gland (SG) groups as variables with each having 14,447 observations corresponding to the expression values of all expressed sequence tags (ESTs) included in the microarray design. The cumulative proportion of variance was 98.4%. Different sets of infection regulated genes were plotted to look for possible correlations between the tissue-specific gene regulation and the tissue-specific gene expression level (MG/SG ratios). (A) Mapping of all genes in the study (14,447). (B) Up-regulated genes in midgut at day 2 of infection. (C) Down-regulated genes in midgut at day 2 of infection. (D) Up-regulated genes in the salivary gland at day 9 of infection. (E) Down-regulated genes in the salivary gland at day 9 of infection.

PCA is a fundamental tool for multivariate analysis of microarray data that provides a two dimensional visual display of data revealing the genes that are under- or over-expressed in one set of samples relative to another. To determine how *R. microplus* gene expression was distributed in midgut and salivary glands relative to our initial steady state in the tick, PCA was carried out with unfed midgut and unfed salivary gland groups as variables (Fig. 2). This allowed us to determine the gene distribution according to their midgut (MG) to salivary gland (SG) ratios (MG/SG = 1, MG/SG < 1, MG/SG > 1) within the entire set of *R. microplus* genes and compare the MG/SG distribution from the different sets of regulated genes to look for possible correlations between the tissue-specific gene expression level and gene regulation. Most genes (8416; 58%) were equally expressed in the midgut and salivary gland of unfed adult male ticks (Fig. 2A). Fig. 2B shows genes that were up-regulated in the midgut at day 2 of infection, revealing that 57% were even more highly expressed in the salivary gland, a higher trend than observed for the global distribution (23%). This distribution shift was not evident in the set of genes that showed down-regulation at day 2 of infection in the midgut, where the distribution was more in agreement with the general distribution being 55%, 21% and 24% for MG/SG = 1, MG/SG < 1 and MG/SG > 1, respectively (Fig. 2C).

Although most sequences in BmiGI V2.1 lack annotation, we analysed the sets of regulated genes for sequences which had Gene Ontology (GO) annotation available (Ashburner et al., 2000). Examining the regulated genes in the midgut at day 2 of infection revealed that genes that were more highly expressed in salivary gland (MG/SG < 1) contained subsets of genes with related GO annotation (Supplementary Table S1); the up-regulated genes included known salivary gland enzymes and inhibitors (TC24673 and TC18832), genes involved in transcription (TC17455, TC19092, TC24786, TC15538, TC22669, TC23017, TC21304, TC19039, TC19181, TC22569) and transporters (TC21639, TC19722, CV451779, TC24412, TC23253, TC17276, TC23935, TC21753, TC21969, TC21073, TC23311, CV442574, TC15918) (Supplementary Table S1). Among the down-regulated genes (Supplementary Table S1) were several genes involved in translation such as ribosomal proteins and translation factors (CK185526, CV440971, TC22754, TC16079, CV457402, TC20808, TC24012, TC23226, TC23318, CV455568, TC23310, AA257897, TC19338, U92743, TC22523, U92753).

Similarly, analysis of genes regulated in the salivary gland at day 9 of infection showed the analogous phenomenon. Most (79) of the 106 up-regulated genes were actually more highly expressed in the midgut (MG/SG > 1) (Fig. 2D). Comparing this percentage (75%) with that observed in the global distribution it was clear that the number of up-regulated genes in salivary gland at day 9 of infection that are actually more highly expressed in the midgut (MG/SG > 1) is considerably higher than the number that would have been expected (18.7%) from the general distribution. Again this marked shift did not occur in the distribution of the 40 down-regulated genes where the percentages were 60, 30 and 10 for the groups MG/SG = 1, MG/SG < 1 and MG/SG > 1, respectively (Fig. 2E). Analysis of GO annotation in these sets of genes showed that among the up-regulated genes in the salivary gland at day 9 of infection that were actually more highly expressed in the midgut (MG/SG > 1) were some known midgut genes (TC22463, TC19214, NP883645, TC21276) (Table 3). Among the down-regulated genes (Table 3) there were several transporters (CV450818, TC24452, CV455105, TC23196, CK184678).

### 3.5 *Rhipicephalus microplus* differential gene expression upon feeding

**Fig. 3:**
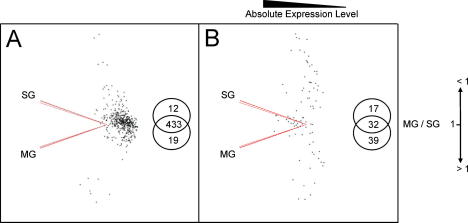
Principal Component Analysis (PCA) of *Rhipicephalus microplus* genes highly regulated upon feeding. PCA with unfed midgut (MG) and salivary gland (SG) groups as variables with each having 14,447 observations corresponding to the expression values of all the expressed sequence tags (ESTs) included in the microarray design. The cumulative proportion of variance was 98.4%. Highly regulated genes (8 ⩽ fold-change ⩽ −8) upon feeding at all time points in MG and SG were plotted to look for possible correlations, if any, between the tissue-specific gene expression level and the magnitude of the gene regulation. (A) Highly up-regulated genes. (B) Highly down-regulated genes.

In contrast to the modest tick response to infection, a dramatic response was observed during feeding with thousands of genes being regulated in the midgut and salivary gland. There were 464 highly up-regulated genes with a fold-change between eight and 293, and 88 highly down-regulated genes with a fold-change between −8 and −164 (Table 2). PCA was carried out with unfed midgut and salivary gland groups and the gene distribution according to their MG/SG ratios (MG/SG = 1, MG/SG < 1, MG/SG > 1) were determined to look for possible correlations between the tissue-specific gene expression level and the magnitude of the gene regulation (Fig. 3). Most of the highly up-regulated genes (433 out of 464) were equivalently expressed between midgut and salivary gland and their absolute expression level tended to be low (Fig. 3A). Ten of 12 highly up-regulated genes with MG/SG < 1 were actually highly up-regulated in the gut, i.e. these genes exhibited a high degree of up-regulation in both midgut and salivary gland. Among this set of genes (Table 4) three IgG-binding protein A sequences (TC20108, TC21571, CV437130) were identified in the BmiGI V2.1 database. Contrary to the observation for the highly up-regulated genes, 37 out of 39 highly down-regulated genes with MG/SG > 1 were primarily highly down-regulated in midgut (Fig. 3B) and all 17 highly down-regulated genes with MG/SG < 1 were also highly down-regulated in salivary gland (Fig. 3B).

**Fig. 4:**
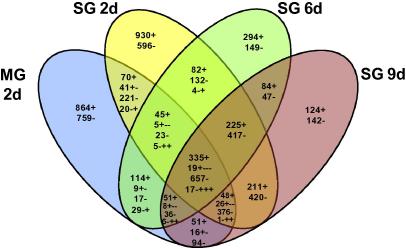
Venn diagram representing *Rhipicephalus microplus* genes regulated by feeding. The number of regulated genes is shown followed by signs which denote the direction of the regulation; the single plus (+) or minus (−) sign indicates up- or down-regulation, respectively, in all groups included in the intersection. Multiple plus (+) and minus (−) signs depict the gene expression regulation observed in the all groups included in the intersection starting from the group labelled ‘MG 2d’ to the group labelled ‘SG 9d’. MG, midgut; SG, salivary glands; d, day.

A Venn diagram (Fig. 4) captures the global distribution of gene regulation. Typically, the salivary gland groups had a good correlation among the genes that were regulated and in the direction of gene regulation, but the genes regulated in the midgut were distinct from the genes regulated in the salivary gland (Fig. 4). There was a rapid and unique response to feeding with 930 up- and 596 down-regulated genes seen only at the day 2 time point. Although there was a massive response with 7,820 genes being regulated in the various treatment groups, there was a relatively small number of co-ordinately regulated genes amongst all treatment groups – 335 up- and 657 down-regulated genes (Fig. 4).

### 3.6 Impact of *A. marginale* infection on tick feeding

The effect of *A. marginale* infection on tick gene response to feeding was evaluated by analysing how the sets of genes that showed regulation upon feeding were regulated upon infection (Table 5). None of the feeding-regulated genes in the salivary gland at days 2 and 6 exhibited additional regulation upon infection despite the fact that the day 2 time point showed the largest number of genes being modulated (1,989 up and 2,937 down) as well as having the highest positive fold-change values (293 and 78). A small percentage of genes regulated by feeding at day 2 in the midgut (7%) and at day 9 in the salivary gland (2%) were affected by infection. The percentage of regulated genes observed in this dataset is similar to that observed for each time point when looking at the response to infection within the whole dataset (Table 2), suggesting that *A. marginale* infection did not have a significant impact on the response to feeding. Interestingly, more genes were differentially regulated by the two treatment parameters than were similarly regulated by the two treatment parameters. For example, 98 genes that were up-regulated upon feeding were down-regulated by infection, and 255 genes that were down-regulated by feeding were up-regulated by infection, whereas 20 or 18 genes were up- or down-regulated, respectively, by both treatments (Table 5).

## 4 Discussion

We tested three hypotheses in this study. The first hypothesis, that the tick transcriptome is organ specific, is accepted based on both the number of genes (>1,600) that are uniquely regulated within the midgut during feeding (Fig. 4) and the presence of regulated genes in the midgut at day 2 of infection when there is a complete absence of regulated genes in the salivary gland (Table 2). This latter difference, at first glance, may appear to be a reflection of the timing of infection: at day 2 the midgut has been colonised by *A. marginale*, while there are very low rates and levels of infection in the salivary gland at this time point (Table 1). However, comparison of the regulated genes in the salivary gland at day 9 of infection with the regulated genes in the midgut at day 2 when infection rates and levels are more similar, shows there is still a large disparity in the numbers of regulated genes in these two tissues (Tables 1 and 2). The organ-specific transcriptomes are consistent with the unique functions these tissues play in transmission – the midgut is composed of highly phagocytic cells which play a role in *A. marginale* uptake, while the salivary gland is highly secretory in nature, necessary for egress of *A. marginale* from the salivary gland into the saliva from where transmission can occur (Agbede and Kemp, 1985; Nunes et al., 2006). We and others have used embryonic tick cell lines to study the pathogen–tick vector interaction (Nelson et al., 2008; Zivkovic et al., 2009; Ramabu et al., 2010; Thepparit et al., 2010). While these in vitro studies have clearly been valuable in enhancing understanding of the interaction, the differences between tick tissues require that, as a minimum, conclusions from embryonic cell line experiments be validated in specific organs and emphasize the importance of deriving cell lines that represent specific, relevant tissues.

The second tested hypothesis that the salivary gland transcriptome is temporally dynamic is supported by a peak of regulated genes at day 2 of feeding (Table 2) and unique sets of regulated genes at each time point (Fig. 4). Ticks secrete a large number of proteins that create a functional feeding site and influence immediate and delayed host responses that would interfere with complete tick feeding (Francischetti et al., 2009). However, the temporal expression of these genes during feeding has not been reported. Three recent publications have reported on the sialotranscriptome of *Rhipicephalus sanguineus*, *Ixodes scapularis* and *Ixodes ricinus* ticks, however these studies appear to have gene discovery as their primary goal and have examined relatively few numbers of genes (<2,000 genes) (Valenzuela et al., 2002; Chmelar et al., 2008; Anatriello et al., 2010). The present study provides evidence that regulation of genes continues throughout the feeding process. For example, TC19720, a salivary gland metalloprotease, is not up-regulated until day 9 of feeding, while CK172486, a metalloprotease inhibitor, is up-regulated by day 2 and remains up-regulated throughout the feeding period. Similar to the importance of the tissue-specific responses noted above, the temporal dynamics of the tick vector transcriptome illustrates the importance of examining the pathogen–vector interaction during tick feeding.

Accepting or rejecting the third hypothesis, that *A. marginale* colonisation does not significantly modulate the tick salivary gland transcriptome, is more nuanced. The presence of genes regulated upon infection does indicate that there is specific modulation of the salivary gland transcriptome. However, the very small number of genes that are regulated upon infection (1–6%) compared with feeding (19–34%; Table 2), as well as the small percentage of feeding regulated genes that are regulated by infection (2–12%; Table 5), supports that the modulation is relatively minor. This conclusion is supported by a recent report using suppression subtractive hybridisation (SSH) that found only 43 and 56 genes to be up- or down-regulated, respectively, in *R. microplus* salivary glands in response to *A. marginale* infection (Zivkovic et al., 2010). This minimal effect on transcription is consistent with observational studies in which *A. marginale* infection has no demonstrable effect on tick activity or survival. This adaptation in the pathogen–vector interaction is in contrast to other pathogens, such as the apicomplexan *Babesia bovis*, which exerts a visibly detrimental effect on its vector (Ouhelli et al., 1987).

The vast majority of *R. microplus* genes regulated in response to infection were hypothetical or of unknown molecular function. Just six of the 106 genes found to be up-regulated at day 9 had GO annotation associated with them, with four having roles in binding (TC18678, TC18348, TC21276 and TC24704) and two having catalytic roles (TC19396 and TC23917). Only a single gene that was down-regulated at day 9 had GO annotation corresponding to the molecular function of electron transport (TC15606, Cytochrome B561). Less than 5% of the genes up- or down-regulated in the midgut at day 2 had GO annotation. Of the 496 genes up-regulated in the midgut at day 2, just 16 were classified for their roles, which included DNA/RNA metabolism (six), catalysis (five) and binding (five) (Table 6). Similarly, 18 genes were classified by GO annotation roles amongst the 392 genes found to be down-regulated at day 2 in the midgut, which included DNA/RNA processing/ribosomal proteins (seven), binding (six), catalysis (three) and structural proteins (two) (Table 6). Translational effects are expected as five ribosomal proteins were found to be down-regulated. Notably, no identical genes were found to be regulated when the SSH results were compared with the present microarray data (Zivkovic et al., 2010). This likely reflects biological differences between the two studies as well as the different methodology: in the SSH study, *R. microplus* larvae were applied and allowed to feed on the calves for 21 days before collection, while the present study used adult male ticks fed for shorter periods. Interestingly TC24862, found to be up-regulated in the microarray data, was identified as a significant match to the SSH-detected GO496186, having 61% nucleotide identity. These sequences are annotated differently, with TC24682 identified as having identity to the ras-like GTPase superfamily and GO496186 being a hypothetical protein.

Overall, the BmiGI V2.1 used for the array contains ∼60% hypothetical genes, reflecting the unique phylogenetic position of these arthropods and the lack of exploration within this branch of life (Wang et al., 2007). Searching unknown genes of interest against the recently available *I. scapularis* data at VectorBase did not reveal additional informative annotation (Hill and Wikel, 2005). The genes regulated by *A. marginale* infection exhibited a higher representation of hypothetical genes than might be expected, suggesting that although the number of regulated genes is small, *A. marginale* infection exerts an effect upon a novel set of *R. microplus* genes that may facilitate pathogen transmission yet not affect tick viability. These hypothetical genes would be candidates for RNA interference (RNAi) experiments, which have been widely adopted to assess the function of tick genes (de la Fuente et al., 2007; Bastos et al., 2009, 2010; Dai et al., 2010). Interestingly, stress response genes and defensins were not up-regulated in response to infection, consistent with evidence that *A. marginale* is well-adapted to colonisation within and transmission by *R. microplus*.

While functional genomic studies of tick–pathogen interactions are in their infancy, this arena is slightly more advanced for mosquito vectors, with two papers assessing the impact of a pathogen on vector transcription. The first study examined *Plasmodium* invasion of the midgut of *Anopheles* mosquitoes and, consistent with studies showing killing of the pathogen in the midgut (Dong et al., 2006), a complex response was mounted including genes involved in cytoskeleton remodelling and humoral immunity (Vlachou et al., 2005). The second study profiled the transcriptional response of *Culex quinquefasciatus* salivary glands to infection with West Nile virus (WNV) and also found a complex response that included genes involved in defence (immunomodulatory and detoxification) and many cellular processes (Girard et al., 2010). A difference between the previous and current studies profiling the tick response to *A. marginale* infection is the sheer number of genes that were found to be differentially regulated: the mosquito studies each had ∼10% of the genes assayed being differentially regulated, while the results from our study, which assayed the largest number of genes, indicated only ∼1–5% of the genes assayed being differentially regulated. In contrast to the present study, both mosquito studies indicate that immunomodulatory genes and genes involved in defense are up-regulated by the pathogens. These findings are consistent with differences in the infection biology of these pathogens in their respective vectors; both *Plasmodium* and WNV have profound effects on their vectors, while *A. marginale* does not.

The study presented here is, to our knowledge, the first global analysis of tick vector response to pathogen infection over time. Our findings demonstrate that *A. marginale* is well adapted to life within the arthropod vector, exerting a minimal effect at the level of gene transcription. Affected genes are primarily hypothetical, but include genes with roles in binding, catalysis and DNA/RNA metabolism. Our results indicate that the transcriptional effect may be amplified by a reduction in translation to create a more profoundly altered proteome. The dataset provided here will be a resource for investigators examining the vector–pathogen interface.

## Figures and Tables

**Table 1:** Tick group size and *Anaplasma marginale* infection rates and levels.

Tissue	Treatment	Uninfected		Infected (*Anaplasma marginale*)			
		Group^a^	Single^b^	Group^a^	Single^b^	Rate^c^	Level^d^
MG	Unfed	90	25				
MG	2d Fed	90	17	90	18	89	1.78 × 10^4(±0.23)^
SG	Unfed	90	25				
SG	2d Fed	90	17	90	18	6	2.55 × 10^2^^e^
SG	6d Fed	90	20	90	20	40	6.53 × 10^3(±1.69)^
SG	9d Fed	90	18	90	17	65	5.94 × 10^5(±0.89)^

a Each treatment group was analysed in triplicate and each replicate consisted of 90 ticks; d, days.

b Number of dissected midgut (MG) or salivary gland (SG) pairs from individual ticks.

c Infection rate = (number positive/number analysed) × 100.

d Infection level = mean number of bacteria/organ (±S.D.).

e S.D. could not be calculated for this single infected tick.

**Table 2:** Summary of *Rhipicephalus microplus* gene regulation levels upon *Anaplasma marginale* infection or feeding.

	Treatment		*Anaplasma marginale* Infection				Blood feeding			
	Tissue		Midgut	Salivary glands			Midgut	Salivary glands		
	Time point		2 d	2 d	6 d	9 d	2 d	2 d	6d	9d
Fold-change^b^	Up	Highest^c^	5.3	1	1	10.0	29.4	292.6	77.7	25.8
		256–512						1		
		128–256						1		
		64–128						8	1	
		32–64						63	6	
		16–32					5	114	17	7
		8–16				1	17	222	18	17
		4–8	5			20	99	259	50	88
		2–4	105			50	614	581	374	401
		1–2	386			35	967	740	825	640
		1	13,559	14,447	14,447	14,301	10,484	9,521	11,637	11,036
	Down^e^	1–2	138			9	1,212	1,759	846	1,286
		2–4	209			31	885	1,069	572	826
		4–8	40				120	92	72	105
		8–16	5				34	14	20	24
		16–32					8	2	5	12
		32–64					1		3	3
		64–128						1	1	2
		128–256					1			
		Lowest^d^	11.6	1	1	3.8	163.7	117.7	86.8	78.2

Regulation	Up		496	0	0	106	1,702	1,989	1,291	1,153
	No change		13,559	14,447	14,447	14,301	10,484	9,521	11,637	11,036
	Down		392	0	0	40	2,261	2,937	1,519	2,258

Total^a^			14,447	14,447	14,447	14,447	14,447	14,447	14,447	14,447
% Regulated genes			6	0	0	1	27	34	19	24

a Total number of genes corresponds to the 14,447 *Rhipicephalus microplus* sequences used on the microarray.

b Data are given as fold-change values when comparing the treated groups and their respective control groups; infected versus uninfected and fed versus unfed; (*q* < 0.05) determined by eBayes method with the Benjamini & Hochberg’s method False Discovery Rate correction procedure.

c Corresponds to the fold-change of the most up-regulated gene under each given condition.

d Corresponds to the fold-change of the most down-regulated gene under each given condition.

e The sign and direction of these fold-change values are negative, d, days.

**Table 3:** Differentially expressed genes in tick salivary gland at day (d) 9 of *Anaplasma marginale* infection with midgut to salivary gland ratios (MG/SG) > 1.

^a^ Expressed Sequence Tag (EST) ID based on the *Rhipicephalus microplus* EST database, *R. microplus* Gene Index Version 2.1 (BmiG.V2.1). ^b^ Reports the accession number, the functional gene name and the species with the highest BLAST hit as reported in BmiGI V2.1. ^c^ Fold-change is the Robust Multi-array Average (RMA) normalised ratio (log2(9 d infected SG/9 d fed SG)). Positive and negative values correspond to up and down regulated genes, respectively; d, day. ^d^ MG/SG is the RMA normalised ratio (log2(unfed MG/unfed SG)). Values of MG/SG > 1, MG/SG < 1 and MG/SG = 1 correspond to genes that are more expressed in midgut, more expressed in salivary gland or equally expressed in both tissues, respectively. ^e^ Shaded blocks indicate groups of functionally related genes.

**Table 4:** Highly up-regulated *Rhipicephalus microplus* genes upon feeding, with midgut to salivary gland ratios (MG/SG) < 1.

^a^ Expressed Sequence Tag (EST) ID based on the *Rhipicephalus microplus* EST database, *R. microplus* Gene Index Version 2.1. (BmiGI V2.1). ^b^ Reports the accession number, the functional gene name and the species with the highest BLAST hit as reported in BmiGI V2.1. ^c^ Fold-change is the Robust Multi-array Average (RMA) normalised ratio (log2(fed group/unfed control group)). ^d^ MG/SG is the RMA normalised ratio (log2(unfed MG/unfed SG)). Values of MG/SG < 1 correspond to genes that are more highly expressed in salivary gland than in midgut. ^e^ Shaded block indicates groups of functionally related genes.

**Table 5:** Impact of *Anaplasma marginale* infection on tick feeding.

Gene regulation		Treatment group			
Upon feeding	Upon infection	2 d MG	2 d SG	6 d SG	9 d SG
Up	Up	20	0	0	6
Up	–	1,584	1,989	1,291	1,131
Up	Down	98	0	0	16
Total Up		1,702	1,989	1,291	1,153
	% Regulated genes^a^	7	0	0	2
Highest fold-change		29	293	78	26
	Highest fold-change	4	1	1	6
Down	Up	255	0	0	42
Down	–	1,988	2,937	1,519	2,214
Down	Down	18	0	0	2
Total Down		2,261	2,937	1,519	2,258
	% Regulated genes^b^	12	0	0	2
Lowest fold-change		−164	−118	−87	−78
	Lowest fold-change	−8	−1	−1	−3

MG, midgut; SG, salivary glands; d, day.

a Percentage of up-regulated genes upon feeding that are also regulated upon infection.

b Percentage of down-regulated genes upon feeding that are also regulated upon infection.

**Table 6:** Genes regulated by infection at day 2 in the *Rhipicephalus microplus* midgut with gene ontology (GO) annotation.

EST ID^a^	Description^b^	GO role	Regulation up/down
TC19495	Q6WCQ5, Block of proliferation protein	DNA/RNA processing	Up
TC23639	Q6PHL7, Tceb2-prov protein	DNA/RNA processing	Up
TC16738	Q99PV0, Pre-mRNA-processing-splicing factor 8		
	DNA/RNA processing	Up	
TC15811	O14776, Transcription elongation regulator 1	DNA/RNA processing	Up
TC24409	Q7PYX5, AGAP011626-PA;	DNA/RNA processing	Up
TC21812	A7KP99, cAMP response element modulator tau	DNA/RNA processing	Up
TC24733	A6NK09, Uncharacterised protein PCID2;	Catalytic	Up
TC17626	Q9Z0H0-2, cdc7-related protein kinase	Catalytic	Up
TC20025	A2V728, Glutamine: fructose-6-phosphate aminotransferase	Catalytic	Up
TC23001	UPI000153886B, AGAP000577-PA, AMP deaminase family	Catalytic	Up
TC21253	Q7PYX5, AGAP011626-PA	Catalytic	Up
TC20184	P35220, Catenin alpha	Binding	Up
TC18727	Q7PRN5, AGAP000812-PA	Binding	Up
TC22974	Q0HA38, Tetratricopeptide repeat protein 21B	Binding	Up
TC24786	Q17PA5, Histone acetyltransferase gcn5	Binding	Up
TC17362	Q9VZL3, CG10849-PA	Binding	Up
TC15813	UPI00003AAB34, Pre-mRNA-splicing factor RBM22 (RNA-binding motif protein 22)	DNA/RNA processing	Down
TC22419	Q5ZHP3, UPF0468 protein C16orf80 homologue	DNA/RNA processing	Down
TC22523	Q86FP6, 40S ribosomal protein S12	Ribosome	Down
TC19338	Q4PM43, Ribosomal protein L15	Ribosome	Down
TC23226	A6N9R2, Ribosomal protein S18	Ribosome	Down
TC23318	Q4PM82, Ribosomal protein S25	Ribosome	Down
TC24012	Q4PM16, 60S ribosomal protein L23	Ribosome	Down
TC17189	A4FV74, MGC143355 protein	Binding	Down
TC17662	Q6WNX3, Ferritin	Binding	Down
TC19571	Q9NZD8, Maspardin	Binding	Down
TC22376	O01679, Rad51 homologue	Binding	Down
TC23016	Q6PV61, Histone H2A	Binding	Down
TC24038	Q178J6, Beta chain spectrin	Binding	Down
TC22382	Q7PXY3, NADH-ubiquinone oxidoreductase 75 kDa subunit	Catalytic	Down
TC16905	A8MX47, Uncharacterised protein PEPD	Catalytic	Down
TC17450	Q8UWJ5, CDH1-D	Catalytic	Down
TC17203	Q16G00, Tetraspanin 97e	Cellular component	Down
TC21978	Q09JH3, Transmembrane protein 14C	Cellular component	Down

a Expressed Sequence Tag (EST) ID based on the *Rhipicephalus microplus* EST database, *R. microplus* Gene Index Version 2.1 (BmiGI V2.1).

b Reports the accession number and the functional gene name as reported in BmiGI V2.1.

## Appendix A Supplementary data

Supplementary Table 1 Differentially expressed genes in midgut at day 2 of infection with midgut (MG) to salivary gland (SG) ratios (MG/SG < 1).
