# Supplementary material for: Temporal characterisation of the organ-specific Rhipicephalusmicroplus transcriptional response to Anaplasma marginale infection
Source: Int J Parasitol. 2011 Jul;41(8):851–60. doi: 10.1016/j.ijpara.2011.03.003 (PMC3114074; doi:10.1016/j.ijpara.2011.03.003)
Supplement: Supplementary Table 1 — Differentially expressed genes in midgut at day 2 of infection with midgut (MG) to salivary gland (SG) ratios (MG/SG < 1). [file mmc1.doc]

**Supplementary Table S1.** Differentially expressed genes in midgut at day 2 of infection with midgut (MG) to salivary gland (SG) ratios (MG/SG<1).

|  | |  |  |
| --- | --- | --- | --- |
| **EST ID^a^** | **Description^b^** | **Fold change^c^** | **MG/SG^d^** |
| **Up-regulated genes in midgut at day 2 upon infection with MG/SG<1** | | | |
| TC24673 | Q45R47, Salivary gland metalloprotease (*Rhipicephalus microplus*)^e^ | 2.782 | 0.01 |
| CK188901 | Q1ZZW9, Metalloproteinase (*Rhipicephalus haemaphysaloides*) | 2.928 | 0.01 |
| TC20070 | Q9BLH1, Neutral endopeptidase 24.11 (*Bombyx mori*) | 2.967 | 0.01 |
| TC18832 | Q5VJG2, Thrombin inhibitor (*Amblyomma hebraeum*) | 2.042 | 0.02 |
| TC24037 | Q12YU9, DNA repair protein radC homolog (*Methanococcoides burtonii* DSM 6242) | 2.170 | 0.26 |
| TC18371 | Q8KQH5, DNA polymerase III epsilon subunit (Candidatus *Tremblaya princeps*) | 1.669 | 0.37 |
| TC17455 | Q1W1Q1, DNA-directed RNA polymerase (*Tricholomopsis decora*) | 1.994 | 0.39 |
| TC19092 | A0Q979, Transcriptional regulator, TetR family protein (*Mycobacterium avium* 104) | 2.479 | 0.01 |
| TC24786 | Q17PA5, Histone acetyltransferase gcn5 (*Aedes aegypti*) | 1.884 | 0.77 |
| TC15538 | Q7SX95, Max-interacting transcriptional repressor MAD3 (*Danio rerio*) | 1.875 | 0.51 |
| TC22669 | A1FFW9, Transcriptional regulator (*Pseudomonas putida* W619) | 1.711 | 0.21 |
| TC23017 | A0L9Z7, Transcriptional regulator, MucR family (*Magnetococcus* sp. MC-1) | 1.656 | 0.28 |
| TC21304 | Q2J5T9, Transcriptional regulator, BadM/Rrf2 family (*Frankia* sp. CcI3) | 1.577 | 0.10 |
| TC19039 | Q5Z4Y8, Regulator of nonsense transcripts 1-like (*Oryza sativa* Japonica Group) | 1.563 | 0.71 |
| TC19181 | UPI00015520DD, SNF2 histone linker PHD RING helicase (*Rattus norvegicus*) | 1.420 | 0.69 |
| TC22569 | P58797, Nuclear RNA export factor 1 (*Coturnix japonica*) | 1.481 | 0.71 |
| TC20066 | Q4E955, Queuine tRNA-ribosyltransferase (*Wolbachia* endosymbiont of Drosophila ananassae) | 1.809 | 0.41 |
| TC20824 | Q4LDR1, BZIP factor splicing isoform (Human T-lymphotropic virus 1) | 1.702 | 0.57 |
| TC21222 | A4RQM0, Ribosomal protein S10, component of cytosolic 80S ribosome and 40S small subunit (*Ostreococcus lucimarinus* CCE9901) | 1.939 | 0.31 |
| TC17289 | A5CC87, 50S ribosomal protein L28 (*Orientia tsutsugamushi* Boryong) | 1.923 | 0.18 |
| CK182821 | A6AD68, Nonribosomal peptide synthetase VibF (*Vibrio cholerae* 623-39) | 1.560 | 0.41 |
| TC21639 | Q99J27, Acetyl-coenzyme A transporter 1 (*Mus musculus*) | 2.973 | 0.05 |
| TC19722 | A0AYV3, Cationic amino acid ABC transporter, periplasmic binding protein precursor (*Burkholderia cenocepacia*) | 2.294 | 0.05 |
| CV451779 | A5VV97, ABC transporter, permease protein (*Brucella ovis* ATCC 25840) | 2.157 | 0.35 |
| TC24412 | Q5WL94, Sugar ABC transporter substrate-binding protein (*Bacillus clausii* KSM-K16) | 1.831 | 0.64 |
| TC23253 | UPI00005F9C60: COG2095, Multiple antibiotic transporter (*Yersinia intermedia* ATCC 29909) | 1.820 | 0.48 |
| TC17276 | UPI00003838A8: COG0531, Amino acid transporters (*Magnetospirillum magnetotacticum* MS-1) | 1.783 | 0.10 |
| TC23935 | Q2UV43, Na+/H+ antiporter (*Aspergillus oryzae*) | 1.740 | 0.47 |
| TC21753 | Q16Z20, Monocarboxylate transporter (*Aedes aegypti*) | 1.736 | 0.59 |
| TC21969 | Q6IMV8, Transposase (*Oryza sativa* Indica Group) | 1.674 | 0.03 |
| TC21073 | Q0YSA6, ABC transporter, substrate-binding protein, aliphatic sulphonates precursor (*Chlorobium ferrooxidans* DSM 13031) | 1.640 | 0.50 |
| TC23311 | A1VZ32, Potassium-transporting ATPase, B subunit, degenerate (*Campylobacter jejuni* subsp. Jejuni) | 1.603 | 0.64 |
| CV442574 | A2V1T8, Transporter hydrophobe/amphiphile efflux-1 (HAE1) family (*Shewanella putrefaciens*) | 1.513 | 0.31 |
| TC15918 | Q220B7, Binding-protein-dependent transport systems inner membrane component precursor (*Rhodoferax ferrireducens* T118) | 1.451 | 0.60 |
| TC21268 | Q6SHZ5, Uroporphyrinogen-III synthase (uncultured marine bacterium 106) | 1.959 | 0.38 |
| CV445099 | A7NHE4 Cluster, Polysaccharide biosynthesis protein (*Roseiflexus castenholzii* DSM 13941) | 1.816 | 0.63 |
| TC22755 | A8DFP0, Precorrin-3B synthase (*Methylobacterium populi* BJ001) | 1.533 | 0.53 |
| TC22721 | A7LFZ7, 5'-nucleotidase (*Ixodes scapularis*) | 2.164 | 0.01 |
| CV450018 | A5IE95, Ribonuclease E (*Legionella pneumophila* str. Corby) | 2.099 | 0.06 |
| TC19799 | Q9HRQ5, Endonuclease III (*Halobacterium salinarum*) | 1.482 | 0.10 |
| TC20106 | Q3D136, Glutamine amidotransferase (*Streptococcus agalactiae*) | 3.017 | 0.04 |
| TC16662 | A5P4Y1, Peptidase S1 and S6, chymotrypsin/Hap precursor (*Methylobacterium* sp. 4-46) | 2.678 | 0.08 |
| CV442271 | Q6CFH3, Serine/threonine protein phosphatase (*Yarrowia lipolytica*) | 2.390 | 0.17 |
| TC19556 | A2PZ96, Acetylcholinesterase (*Pediculus humanus corporis*) | 2.387 | 0.06 |
| CV436064 | A6WBT8, Monooxygenase FAD-binding (*Kineococcus radiotolerans* SRS30216) | 2.349 | 0.02 |
| TC19172 | Q0FLQ9, Peptidoglycan transglycosylase (*Roseovarius* sp. HTCC2601) | 2.192 | 0.15 |
| CV451780 | Q196P0, NADH-ubiquinone oxidoreductase chain 6 (*Mussa angulosa*) | 2.157 | 0.25 |
| CK192538 | A8QKT1, NADH dehydrogenase subunit 4 (*Leptodeira septentrionalis* polysticta) | 1.943 | 0.02 |
| TC16578 | UPI000065CD0F, Angiotensin-converting enzyme, somatic isoform precursor, CD143 antigen (*Takifugu rubripes*) | 1.884 | 0.49 |
| TC21089 | A7DX11, Parvulin-type peptidyl prolyl cis/trans isomerase (*Lotus japonicus*) | 1.751 | 0.11 |
| TC25007 | Q1WMH0, NADH-ubiquinone oxidoreductase chain 2 (*Stenocercus empetrus*) | 1.714 | 0.42 |
| CV441163 | A4FHU8, Flavin oxidoreductase/NADH oxidase (*Saccharopolyspora erythraea* NRRL 2338) | 1.671 | 0.30 |
| TC20025 | A2V728, Glutamine: fructose-6-phosphate aminotransferase (*Haemaphysalis longicornis*) | 1.648 | 0.31 |
| TC23699 | A5V6V4, Alcohol dehydrogenase, zinc-binding domain protein (*Sphingomonas wittichii* RW1) | 1.623 | 0.52 |
| CV451727 | Q173X0, UDP-glucose 4-epimerase (Aedes aegypti) | 1.577 | 0.43 |
| TC17571 | A6Y656,SAM-dependent methyltransferase (*Vibrio cholerae* RC385) | 1.569 | 0.40 |
| TC19542 | A6M0M5, Aldehyde dehydrogenase (*Clostridium beijerinckii* NCIMB 8052) | 1.558 | 0.69 |
| TC23962 | Q35980, NADH-ubiquinone oxidoreductase (*Triticum aestivum*) | 1.539 | 0.49 |
| TC22687 | A3TFT7, N-acetylglucosamine kinase-like protein (*Janibacter* sp. HTCC2649) | 1.517 | 0.54 |
| CV436286 | A8LY18, Dihydroorotate oxidase (*Salinispora arenicola* CNS-205) | 1.504 | 0.74 |
| TC19562 | A7E3M3, Predicted dual oxidase (*Apis mellifera*) | 1.468 | 0.74 |
| CK183753 | A5PHP4, GDP-4-dehydro-6-deoxy-D-mannose epimerase/reductase (*Trypanosoma brucei* brucei) | 1.437 | 0.31 |
| TC18786 | Q14669, Probable E3 ubiquitin-protein ligase TRIP12 (*Homo sapiens*) | 1.375 | 0.55 |
| CV446023 | A8AZ04, Membrane protein (*Streptococcus gordonii* str. Challis substr. CH1) | 2.678 | 0.07 |
| TC19633 | UPI000060FF99, Membrane metallo-endopeptidase-like 1 (2)(*Gallus gallus*) | 2.250 | 0.02 |
| TC18015 | Q97I03, Predicted membrane protein (*Clostridium acetobutylicum*) | 2.004 | 0.25 |
| CV450437 | A4GDJ5, IMV membrane protein (Vaccinia virus) | 1.692 | 0.36 |
| CK187008 | Q3MKD6, Phytochrome C (*Maihuenia patagonica*) | 1.618 | 0.66 |
| CV440502 | A7U490, Cytochrome c oxidase subunit 1 (*Camponotus* sp. MON) | 1.395 | 0.48 |
| CV457184 | Q2LRB9, ATP synthase C chain (*Syntrophus aciditrophicus* SB) | 1.709 | 0.66 |
| TC16554 | Q5A5F0, Possible mitochondrial Complex I, subunit 1 (*Candida albicans*) | 1.429 | 0.52 |
| TC24946 | UPI0000E20A39, mitochondrial ferritin (*Pan troglodytes*) | 1.686 | 0.26 |
| TC24445 | Q41092, Cold stress protein (*Poncirus trifoliata*) | 2.302 | 0.17 |
| CV446170 | UPI000019BF53, small inducible cytokine subfamily A (Cys-Cys), member 17 (*Rattus norvegicus*) | 1.937 | 0.25 |
| TC15373 | O42445, CXC chemokine receptor (*Oncorhynchus mykiss*) | 1.693 | 0.33 |
| CK182938 | Q8PPI1, Type II secretion system protein N (*Xanthomonas axonopodis* pv. Citri) | 1.725 | 0.27 |
| TC23209 | A4XWF6, General secretion pathway M protein (*Pseudomonas mendocina* ymp) | 1.509 | 0.52 |
| CV455463 | A1K5U6, Sensor protein (*Azoarcus* sp. BH72) | 2.305 | 0.02 |
| TC21192 | Q18IX6, Sensor protein (*Haloquadratum walsbyi* DSM 16790) | 1.648 | 0.02 |
| TC22682 | P95194, TWO COMPONENT SENSOR HISTIDINE KINASE DEVS (*Mycobacterium tuberculosis*) | 1.488 | 0.63 |
| TC23977 | A6Q7R3, Acriflavin resistance protein, AcrA/AcrE family (*Sulfurovum* sp. NBC37-1) | 3.816 | 0.28 |
| CK180464 | Q216Y8, Fusaric acid resistance protein conserved region (*Rhodopseudomonas palustris* BisB18) | 1.895 | 0.29 |
| TC23697 | A4XN58, Spore germination protein precursor (*Caldicellulosiruptor saccharolyticus* DSM 8903) | 3.063 | 0.06 |
| TC22836 | Q9RNE1, Spore germination protein GerYB (*Bacillus anthracis*) | 2.199 | 0.11 |
| TC17436 | A1CQG1, GPI anchored serine-threonine rich protein (*Aspergillus clavatus*) | 2.866 | 0.29 |
| TC24617 | A1CFZ7, Conserved glycine-rich protein (*Aspergillus clavatus*) | 2.032 | 0.02 |
| CK180748 | UPI00015AA242, zinc finger, FYVE domain containing 28 (*Mus musculus*) | 2.387 | 0.16 |
| TC20764 | UPI0000661210, Homolog of Homo sapiens \Zinc finger protein 239 (*Takifugu rubripes*) | 1.622 | 0.44 |
| CK188188 | UPI0000DB6A6A, zinc finger protein 457 (*Mus musculus*) | 1.427 | 0.68 |
|  |  |  |  |
| **Down-regulated genes in midgut at day 2 upon infection with MG/SG<1** | |  |  |
|  |  |  |  |
| TC24019 | Q3JSE4, Cobalamin biosynthesis protein CbiD (*Burkholderia pseudomallei* 1710b) | -2.447 | 0.53 |
| TC22566 | Q2T620, Transcriptional regulator, DeoR family (*Burkholderia thailandensis* E264) | -1.630 | 0.43 |
| TC16720 | Q0K348, Transcriptional regulator, Fur-family (*Ralstonia eutropha* H16) | -5.748 | 0.25 |
| CK185526 | A1L3S3, RNA binding motif protein 4 (*Mus musculus*) | -2.167 | 0.54 |
| CV440971 | Q4TTE0, Eukaryotic translation initiation factor 2 alpha subunit (*Bombyx mori*) | -1.463 | 0.62 |
| TC22754 | Q8TYV3, 7-cyano-7-deazaguanine tRNA-ribosyltransferase (*Methanopyrus kandleri*) | -1.840 | 0.48 |
| TC16079 | Q47TZ9, tRNA-adenosine deaminase (*Thermobifida fusca* YX) | -2.398 | 0.29 |
| CV457402 | Q962T1, 60S ribosomal protein L32 (*Spodoptera frugiperda*) | -1.421 | 0.67 |
| TC20808 | Q4PM06, 60S ribosomal protein L14 (*Ixodes scapularis*) | -1.497 | 0.56 |
| TC24012 | Q4PM16, 60S ribosomal protein L23 (*Ixodes scapularis*) | -1.522 | 0.67 |
| TC23226 | A6N9R2, Ribosomal protein S18 (*Ornithodoros parkeri*) | -1.543 | 0.59 |
| TC23318 | Q4PM82, Ribosomal protein S25 (*Ixodes scapularis*) | -1.576 | 0.65 |
| CV455568 | A9QQ28, 60S ribosomal protein L13A (*Lycosa singoriensis*) | -1.587 | 0.57 |
| TC23310 | Q86G64, 40S ribosomal protein S5 (*Dermacentor variabilis*) | -1.619 | 0.64 |
| AA257897 | A6N9L8, 60s ribosomal protein L24 (*Ornithodoros parkeri*) | -1.655 | 0.63 |
| TC19338 | Q4PM43, Ribosomal protein L15 (*Ixodes scapularis*) | -1.699 | 0.57 |
| U92743 | A6N9M1, 40S ribosomal protein S2/30S (*Ornithodoros parkeri*) | -1.703 | 0.62 |
| TC22523 | Q86FP6, 40S ribosomal protein S12 (*Dermacentor variabilis*) | -1.745 | 0.53 |
| U92753 | Q4PM08, Ribosomal protein L40 (*Ixodes scapularis*) | -1.820 | 0.60 |
| TC23752 | A7HAN1, Molybdate ABC transporter, inner membrane subunit (*Anaeromyxobacter* sp. Fw109-5) | -2.261 | 0.53 |
| CK181242 | A0NMQ8, 2-isopropylmalate synthas (*Labrenzia aggregata* IAM 12614) | -3.681 | 0.44 |
| CV451516 | A1S8S5, Apolipoprotein N-acyltransferase precursor (*Shewanella amazonensis* SB2B) | -1.781 | 0.46 |
| TC16555 | Q1D6J9, 1,4-dihydroxy-2-naphthoate octaprenyltransferase (*Myxococcus xanthus* DK 1622) | -1.986 | 0.60 |
| TC18142 | A3NMX1, Acetyltransferase, GNAT family (*Burkholderia pseudomallei* 668) | -2.008 | 0.67 |
| TC22857 | P18869, 3-isopropylmalate dehydrogenase (*Schizosaccharomyces pombe*) | -2.844 | 0.39 |
| TC24394 | UPI00003828B3: COG5446, Predicted integral membrane protein (*Magnetospirillum magnetotacticum* MS-1) | -1.853 | 0.57 |
| TC20931 | UPI00006607AC, Transmembrane gamma-carboxyglutamic acid protein 3 precursor (*Takifugu rubripes*) | -2.224 | 0.62 |
| CV453217 | Q665E9: UPF0191 membrane protein YPTB3569 (*Yersinia pseudotuberculosis*) | -2.563 | 0.55 |
| TC22726 | UPI000065D5E7, Zinc finger UBR1-type protein 1 (Retinoblastoma-associated factor of 600 kDa) (*Takifugu rubripes*) | -2.789 | 0.26 |

^a^ Expressed sequence tag (EST) ID based on *Rhipicephalus microplus* EST database, *R. microplus* Gene Index Version 2.1 (BmiGI).

^b^ Lists the accession number, the functional gene name and the species with the highest BLAST hit as reported in BmiGI V2.1.

^c^ Fold change is the Robust Multi-array Average (RMA) normalized ratio (log2(2 day infected MG/2 day fed MG)). Positive and negative values correspond to up- and down-regulated genes, respectively. ^d^ MG/SG is the RMA normalized ratio (log2(unfed MG/unfed SG)). Values of MG/SG<1 correspond to genes that are more highly expressed in SG.

^e^ Shaded blocks indicate groups of functionally related genes.
